# Associations of Body-Size and Estimated Body-Composition Indicators with Body-Shape Concerns and Eating Disorder-Related Traits in Adolescents: A School-Based Cross-Sectional Study

**DOI:** 10.3390/nu18203360

**Published:** 2026-10-14

**Authors:** Dolores Escrivá, Esther Moreno-Latorre, Inmaculada Benet, Carlos Barrios

**Affiliations:** 1 Nursing Department, School of Medicine and Health Sciences, Valencia Catholic University, 46007 Valencia, Spain; 2 School of Medicine and Health Sciences, Institute for Research on Musculoskeletal Disorders, Catholic University of Valencia San Vicente Mártir, 46001 Valencia, Spain; carlos.barrios@ucv.es; 3 School of Teacher Training and Educational Sciences, Valencia Catholic University, 46110 Godella, Spain; esther.moreno@ucv.es; 4 Surgical Nursing Division, Valencia Clinic Hospital, 46010 Valencia, Spain; inmabenet@yahoo.es

**Keywords:** adolescents, body composition, body image, eating disorders, EDI-3, Body Shape Questionnaire, Drive for Thinness, Mediterranean population

## Abstract

**Background/Objectives:** Adolescence is a critical period for the development of body-shape concerns and eating disorder-related traits. This study examined sex differences and associations among anthropometric and estimated body-composition indicators, body-shape concerns, and eating disorder-related traits in adolescents. **Methods:** This school-based cross-sectional study included a convenience sample of 723 adolescents aged 11–17 years (404 boys and 319 girls) from three secondary schools in Valencia, Spain. Anthropometric and skinfold measurements were obtained, and participants completed the Body Shape Questionnaire (BSQ) and the Drive for Thinness, Bulimia, and Body Dissatisfaction subscales of the Eating Disorder Inventory-3 Referral Form. Between-sex comparisons, sex-stratified Spearman correlations, and multivariable linear regression models were performed. **Results:** Girls had higher estimated body-fat percentages than boys (19.29% vs. 11.95%; *p* < 0.001), as well as higher BSQ (62.23 ± 26.71 vs. 56.62 ± 27.19; *p* < 0.001) and Drive for Thinness scores (11.43 ± 6.55 vs. 8.36 ± 6.26; *p* < 0.001). No significant sex differences were observed for Bulimia or Body Dissatisfaction. Sex-stratified correlations between anthropometric or estimated body-composition indicators and the psychological outcomes were weak. Although several associations reached nominal significance, none remained statistically significant after false-discovery-rate correction for multiple testing. Nevertheless, the adjusted multivariable analysis continued to show an independent association between body-fat percentage and Drive for Thinness. The sex-stratified correlations should therefore be regarded as exploratory findings requiring replication. **Conclusions:** Girls showed greater body-shape concerns and Drive for Thinness. Body-fat percentage was independently associated with EDI-DT scores after adjustment for age and sex; its association with BSQ scores was of borderline significance with robust standard errors. These cross-sectional findings do not support causal interpretations.

## 1. Introduction

Overweight and obesity, body dissatisfaction, and eating disorder-related symptoms during adolescence are closely interrelated but represent distinct and non-interchangeable dimensions of adolescent health [1,2,3]. Adolescence is a critical developmental period characterized by rapid physical, psychological and social changes. During this stage, body composition changes substantially, and adolescents progressively develop a more complex perception of their own body shape, weight and appearance. These changes may contribute to body image concerns and, in some individuals, to attitudes and behaviors associated with eating disorders [4,5].

Childhood and adolescent overweight and obesity remain major public health concerns worldwide [6]. However, the psychological impact of excess weight during adolescence cannot be interpreted solely through body mass index (BMI) or other anthropometric indicators [7]. Although BMI is widely used to classify weight status in epidemiological studies, it does not distinguish between fat mass and lean mass and therefore provides limited information on body composition [8,9]. This limitation is particularly relevant during puberty, when boys and girls follow markedly different trajectories of body composition development, with a proportionally greater increase in fat mass in girls and in fat-free mass in boys [10].

Body image dissatisfaction is commonly considered an important psychological correlate of overweight and obesity; however, the relationship between objective weight status and subjective body image is complex. Although higher BMI is generally associated with greater body dissatisfaction, considerable discordance exists at the individual level. Adolescents with normal weight may report substantial body dissatisfaction, whereas some adolescents with overweight or obesity may show relatively preserved body satisfaction [11,12,13,14]. Therefore, the assessment of eating disorder-related traits requires instruments that capture psychological dimensions beyond anthropometric status [5,15].

The Body Shape Questionnaire (BSQ) assesses concerns about body shape and dissatisfaction [16], whereas the Eating Disorder Inventory-3 Referral Form (EDI-3 RF) assesses three eating-disorder risk dimensions: Drive for Thinness, Bulimia, and Body Dissatisfaction [17,18]. These constructs are expected to show positive associations, particularly BSQ with EDI-BD, although their magnitude may vary by sex, age, and adiposity.

Previous analyses of the original cohort of 809 participants examined nutritional status and BSQ scores [19], as well as the associations of EDI-3 RF risk subscales with BSQ scores, age, sex, and BMI; the latter analysis found no significant correlation between BSQ and EDI-BD [20]. The present study uses a complete-case subset and examines whether additional anthropometric and estimated body-composition indicators provide information beyond age and sex. Because some variables and participants overlap with those publications, the present analyses and their incremental contribution are described explicitly.

The aim of the present study was to examine sex differences and associations among EDI-3 RF risk-subscale scores, BSQ scores, and anthropometric and estimated body-composition indicators in Mediterranean adolescents. A secondary aim was to quantify the association between BSQ and EDI-BD scores; the study was not designed to test measurement equivalence, agreement, or construct distinctiveness. The prespecified hypotheses were: (i) girls would report higher BSQ and EDI-DT scores; (ii) adiposity indicators would correlate positively with BSQ, EDI-DT, and EDI-BD; and (iii) BSQ would show a positive, at least moderate association with EDI-BD.

## 2. Materials and Methods

### 2.1. Study Design and Participants

This cross-sectional observational study used a convenience sample recruited from three secondary schools in Valencia, Spain. The schools were among those collaborating with the Catholic University of Valencia for the internship of their students in the Education program and were selected after agreeing to participate; therefore, the sampling frame was not population random. Data were collected during the 2018–2019 academic year as part of a broader project assessing anthropometry, estimated body composition, body image, and eating disorder-related traits.

Students aged 11–17 years were eligible if parental written consent and adolescent assent had been obtained, if they engaged in at least 6 h of physical activity per week as part of the regular activity program of the participating schools, and if valid age, sex, anthropometric, BSQ, and EDI-3 RF data were available. The present sample is a subset of the previously reported cohort and therefore retained the same eligibility criteria. Exclusion criteria were age outside the prespecified range, missing questionnaire or anthropometric data required for the relevant analysis, duplicate or unmatched participant identifiers, and values determined to be impossible after source-document verification. Of the 1149 students invited to participate in the original project, 809 participated. Eighty-six participants were excluded from the present complete-case analysis: 19 because of missing, impossible, or unreliable anthropometric data; 20 because of missing or invalid BSQ scores; 46 because incomplete or inadequate EDI-3 RF responses prevented calculation of final subscale scores; and one because the participant was older than 17 years. Of the 20 BSQ exclusions, 16 had missing totals and four had stored totals below the theoretical minimum (30, 20, 20, and 20 points). These four values cannot arise from valid scoring of the 34-item questionnaire, and valid totals could not be reconstructed reliably; they were therefore classified as invalid rather than retained as observed scores. Thus, 723 adolescents were included in the main analyses (Figure 1). All values identified as negative, impossible, or potentially unreliable were checked against the original records. Transcription errors were corrected when the source value could be verified; otherwise, the corresponding value was treated as missing or the participant was excluded when the variable was required for the analysis. No anthropometrically implausible values were knowingly retained.

The present analysis uses a complete-case subset of the original 809-participant cohort. Reference [19] reported nutritional status and BSQ scores, whereas Reference [20] examined EDI-3 RF risk subscales, BSQ, age, sex, and BMI and reported the BSQ–EDI-BD association. The current secondary analysis adds the sum of six skinfolds, equation-derived body-fat and muscle-mass indicators, prespecified multiplicity-adjusted sex-stratified correlations and interaction models. It should therefore not be interpreted as an independent cohort.

The study was conducted in accordance with the Declaration of Helsinki. Parents provided written informed consent and adolescents provided assent. The Health Sciences Research Committee of the School of Doctorate of the Catholic University of Valencia reviewed and approved the original project on 23 May 2012. At that time, the committee did not assign protocol codes.

### 2.2. Anthropometric and Body-Composition Assessment

Anthropometric measurements were obtained by a single trained assessor certified as Level II by the International Society for the Advancement of Kinanthropometry (ISAK), following standardized procedures. All participants were measured without shoes and in minimal clothing. Height was measured to the nearest 0.1 cm (SECA 225, SECA, Hamburg, Germany), and body weight to the nearest 0.1 kg (SECA 861, SECA, Hamburg, Germany). BMI was calculated as kg/m^2^. Six skinfold thicknesses (triceps, subscapular, abdominal, suprailiac, thigh, and calf) were measured in triplicate on the right side of the body with a skinfold caliper (Holtain Ltd., Crymych, UK) following ISAK recommendations [21]. The mean of the three measurements was used. Body-fat percentage (BF%) was estimated from the sum of six skinfolds using the sex-specific Yuhasz equations (boys: BF% = 0.1051 × Σ6 + 2.585; girls: BF% = 0.1548 × Σ6 + 3.580) [22]. These equations were retained because they were prespecified in the original study protocol and use the same six-site ISAK skinfold assessment; they were not selected post hoc as being superior to pediatric equations. The Slaughter equations, which were developed specifically for children and adolescents [23], were not applied retrospectively because they use a different skinfold specification and would reduce comparability with the prior reports from this cohort. Fat mass was calculated as (BF%/100) × body weight, and lean mass as body weight minus fat mass. Bone mass was estimated with the Rocha equation [24] from height and the bistyloid and bicondylar femur diameters, residual mass as 24.1% (boys) or 20.9% (girls) of body weight, and muscle mass as body weight minus fat, bone, and residual masses. Table 1 reports muscle mass in kilograms. 

### 2.3. Body Dissatisfaction Assessment

Body-shape concerns were assessed with the validated Spanish 34-item Body Shape Questionnaire (BSQ) [25]. Each item is scored from 1 to 6, yielding a theoretical total range of 34–204; higher totals indicate greater concern about body shape. In the present analytical sample, the observed range was 34–186. The reliability of the questionnaire in young Spanish populations has been supported, with Cronbach’s alpha values between 0.74 and 0.96 in adaptation studies [26,27]. In this sample, Cronbach’s alpha was 0.71. The BSQ total score was analyzed as a continuous variable, and a score of ≥105 was used for the secondary dichotomous analysis in accordance with the cutoff reported for Spanish adolescents [26,27].

### 2.4. Eating Disorder-Related Traits

Eating disorder-related traits were assessed with the validated Spanish EDI-3 Referral Form (EDI-3 RF), not the full 91-item EDI-3. The EDI-3 RF contains the Drive for Thinness (seven items; possible raw score 0–28), Bulimia (eight items; 0–32), and Body Dissatisfaction (10 items; 0–40) risk subscales. Items were scored according to the manual using the symptomatic 4–3–2–1–0–0 scoring rule; positively keyed EDI-BD items were reverse-scored before the symptomatic scoring transformation [28]. In the present analytical sample, internal consistency was acceptable and similar to that previously reported in the original series (EDI-DT = 0.75, EDI-B = 0.70, and EDI-BD = 0.72) [20]. Higher scores indicate greater endorsement of the corresponding trait. Questionnaire scores were not used to establish a clinical diagnosis.

### 2.5. Statistical Analysis

Analyses were conducted using complete observations for the variables included in each analysis. Sex-specific correlations included 404 boys and 319 girls, except analyses involving relative muscle mass, for which data were available for 403 boys and 319 girls. All multivariable models included 723 participants because complete data were available for their outcomes and prespecified predictors: sex, age, and estimated body-fat percentage. No missing values were imputed.

Sex was treated as a categorical variable (boys as the reference category), while age, anthropometric measures, estimated body-composition indicators, BSQ, and EDI-3 RF scores were analyzed as continuous variables. Continuous data are presented as mean ± SD. Distributions, Q–Q plots, and residual plots were examined. Between-sex comparisons were performed using two-sided Mann–Whitney U tests. Mean differences are reported with 95% confidence intervals estimated using Welch’s method. Standardized between-sex differences were quantified using Cohen’s d and interpreted according to conventional thresholds [29].

The Kolmogorov–Smirnov test with Lilliefors correction (appropriate when *n* ≥ 50, as here) rejected normality for all eight continuous variables (age, BMI, body-fat percentage, relative muscle mass, BSQ, and all three EDI-3 RF subscales; *p* < 0.001 in every case), with positive skew for BSQ (skewness = 1.42) and EDI-B (skewness = 1.67). Therefore, non-parametric Mann–Whitney U tests were used for between-sex comparisons. Spearman correlations with 95% confidence intervals were used to quantify associations of EDI-3 RF scores with BSQ and anthropometric or estimated body-composition indicators; the *p*-values of the 38 sex-stratified correlations were adjusted using the Benjamini–Hochberg procedure (q-values). Four prespecified multiple linear regression models used BSQ, EDI-DT, EDI-B, and EDI-BD as continuous outcomes. Sex, age, and estimated body-fat percentage were entered simultaneously on conceptual grounds. Heteroscedasticity was assessed with the Breusch–Pagan test, and HC3-robust standard errors were used when detected (BSQ, EDI-DT, and EDI-BD models). An elevated BSQ score (≥105 points), based on the conventional cutoff reported for Spanish adolescents [26,27], was analyzed with multivariable logistic regression including the same predictors; calibration was assessed with the Hosmer–Lemeshow test.

Potential effect modification by sex was examined in exploratory sensitivity analyses. Age and body-fat percentage were mean-centered, and the sex × age and sex × body-fat percentage interaction terms were added simultaneously to each of the four adjusted models. Boys were used as the reference category. HC3-robust standard errors were used for the BSQ, EDI-DT and EDI-BD models, consistent with the corresponding main models. Because eight interaction terms were evaluated, their *p*-values were adjusted using the Benjamini–Hochberg procedure. Analyses were conducted in Python (SciPy 1.17, statsmodels 0.15, Austin, TX, USA).

## 3. Results

### 3.1. Sample Characteristics and Sex-Related Differences in Body Composition

Table 1 presents the anthropometric and estimated body-composition indicators by sex. Girls were younger by 0.42 years (*p* = 0.011) and had higher mean estimated body-fat percentage (19.29% vs. 11.95%; d = 1.34; *p* < 0.001) and skinfold sum (101.46 vs. 89.10 mm; d = 0.29; *p* < 0.001). Because body-fat percentage was estimated with sex-specific equations, part of this difference reflects the different equation parameters. Boys had higher mean height (mean difference 2.84 cm; *p* = 0.001), weight (mean difference 3.66 kg; *p* < 0.001), BMI (mean difference 0.66 kg/m^2^; *p* = 0.009) and estimated lean mass (mean difference 6.82 kg; *p* < 0.001). Estimated fat mass was higher in girls (mean difference 3.16 kg; *p* < 0.001). Estimated muscle mass was higher in boys (mean difference 3.57 kg, 95% CI 2.83 to 4.32; d = −0.67; *p* < 0.001).

### 3.2. Sex Differences in Body-Shape Concerns and EDI-3 RF Risk Subscales

Girls had higher BSQ scores than boys (62.23 ± 26.71 vs. 56.62 ± 27.19; mean difference 5.61, 95% CI 1.65 to 9.57; d = 0.21; *p* < 0.001). The small standardized difference is reported descriptively; clinical importance was not assessed (Table 2). Girls also had higher EDI-DT scores than boys (11.43 ± 6.55 vs. 8.36 ± 6.26; mean difference 3.07, 95% CI 2.13 to 4.02; d = 0.48; *p* < 0.001), which was the largest standardized sex difference among the three EDI-3 RF risk subscales examined.

No significant sex differences were found for the Bulimia (*p* = 0.159) or Body Dissatisfaction (*p* = 0.572) subscales, nor in the proportion with elevated BSQ score (≥105 points: 9.1% [95% CI 6.4–12.8] in girls vs. 6.4% [95% CI 4.4–9.3] in boys; χ^2^ with continuity correction = 1.43, *p* = 0.232).

### 3.3. Sex-Stratified Associations Between EDI-3 Subscales, Body-Composition Indicators, and BSQ

Several weak sex-stratified correlations reached nominal statistical significance before adjustment for multiple testing (Table 3). In boys, these included BMI with BSQ (ρ = 0.133, *p* = 0.008, q = 0.095), body-fat percentage and the sum of six skinfolds with Drive for Thinness (both ρ = 0.137, *p* = 0.006, q = 0.095), and relative muscle mass with Drive for Thinness (ρ = −0.105, *p* = 0.035, q = 0.210) and Body Dissatisfaction (ρ = −0.117, *p* = 0.019, q = 0.181). In girls, BMI was positively associated with Drive for Thinness (ρ = 0.112, *p* = 0.046, q = 0.210), whereas relative muscle mass was inversely associated with Drive for Thinness (ρ = −0.126, *p* = 0.024, q = 0.182). None of these correlations remained statistically significant after false-discovery-rate correction. Table 3 uses relative muscle mass, calculated as (muscle mass in kg/body weight in kg) × 100.

No significant correlations were observed between BSQ scores and any EDI-3 RF subscale in either sex. Similarly, none of the anthropometric indicators was significantly associated with the EDI-3 RF Bulimia subscale.

### 3.4. Multivariate Models for BSQ, Drive for Thinness, Bulimia, and Body Dissatisfaction

The interaction analyses provided no evidence that the associations of age or body-fat percentage with the study outcomes differed by sex after correction for multiple testing (Table 4). None of the eight interaction terms reached nominal significance (all *p* ≥ 0.080; all q ≥ 0.421); the smallest *p*-value corresponded to the sex × age interaction for EDI-BD (B = 0.533; 95% CI: −0.063 to 1.128). Adding the interaction terms resulted in only minimal increases in explained variance (ΔR^2^ = 0.0009–0.0082). These findings should therefore be interpreted as exploratory patterns rather than confirmatory evidence of association.

In the BSQ model, age was the only independent correlate (B = 2.107; 95% CI: 1.121 to 3.092; *p* < 0.001); the associations with body-fat percentage (B = 0.426; 95% CI: −0.014 to 0.866; *p* = 0.058) and female sex (B = 3.373; 95% CI: −1.816 to 8.562; *p* = 0.203) did not reach statistical significance with HC3-robust standard errors (Table 5). In the Drive for Thinness model, female sex (B = 1.847; 95% CI: 0.700 to 2.994; *p* = 0.002) and body-fat percentage (B = 0.150; 95% CI: 0.061 to 0.239; *p* < 0.001) were positively and age-inversely (B = −0.295; 95% CI: −0.534 to −0.055; *p* = 0.016) associated with the outcome. Body-fat percentage and sex were not independently associated with Bulimia or Body Dissatisfaction. The Bulimia model reached overall statistical significance (*p* = 0.034) only because of its inverse association with age (B = −0.276; 95% CI: −0.470 to −0.082; *p* = 0.005), and the Body Dissatisfaction model was significant (*p* < 0.001), primarily owing to its inverse association with age (B = −0.748; 95% CI: −1.046 to −0.451; *p* < 0.001). Nevertheless, the explanatory capacity of all models was limited, with adjusted R^2^ values ranging from 0.008 to 0.073. The adjusted models accounted for only a small proportion of the variance in the study outcomes.

In logistic regression, each one-percentage-point increase in %BF was associated with a 5.3% increase in the odds of elevated BSQ (OR = 1.053, 95% CI 1.007–1.100; *p* = 0.023), independently of age (OR = 1.221, 95% CI 1.062–1.404; *p* = 0.005); the association with sex was not statistically significant (OR = 1.073, 95% CI 0.552–2.085; *p* = 0.836). The Hosmer–Lemeshow test showed no evidence of lack of fit (χ^2^ = 11.15, df = 8; *p* = 0.193) (Table 6).

## 4. Discussion

The largest and most robust descriptive finding in the submitted analysis was the higher EDI-DT score in girls. Associations between the EDI-3 RF risk scores and anthropometric or estimated body-composition indicators were small and mainly concerning adiposity. Because this cross-sectional study cannot identify determinants, the results are interpreted only as associations within this convenience sample.

Another interesting result in the current sample was that body-fat percentage was independently associated with EDI-DT scores after adjustment for age and sex, whereas its association with BSQ scores was of borderline significance with robust standard errors and was supported mainly by the logistic model for an elevated BSQ score. BMI does not distinguish fat mass from fat-free mass and may incompletely characterize adiposity during puberty [30,31,32]. The Yuhasz equations were retained because they formed part of the original six-skinfold protocol, not because they are known to outperform pediatric equations. They were developed in athletic populations and were not specifically validated for the full age range of this adolescent sample. Although the Slaughter equations were developed for children and youth [23], replacing the prespecified method post hoc would have changed the measurement definition and reduced comparability with the prior cohort reports. Accordingly, the equation-derived values should be interpreted as estimated anthropometric indicators rather than direct or criterion measures of body composition.

The data do not support restricting eating-disorder risk assessment to adolescents with overweight or obesity. This inference is limited to the observation that anthropometric indicators showed small associations with EDI-3 RF scores in this sample and should not be taken as evidence about diagnostic performance or causation [33,34,35].

From a preventive perspective, anthropometric status and self-reported eating disorder-related traits represent different assessment domains. School-based programs should avoid assuming that normal weight excludes clinically important symptoms, while also recognizing that the present questionnaires are screening measures and not diagnostic instruments [35,36,37]. Anthropometric measures provide valuable information on physical and nutritional status, but they do not fully capture psychological vulnerability related to body image, weight concerns, or disordered eating behaviors [38,39]. The integration of body composition assessment with validated psychological instruments may improve early identification of adolescents at risk and guide preventive educational interventions.

The present complete-case subset adds analyses of the sum of six skinfolds, equation-derived body-composition indicators, and prespecified multivariable and interaction models. Because it overlaps completely with the cohorts in References [19,20], the current study should be interpreted as a secondary analysis rather than an independent cohort. Girls had higher EDI-DT scores, whereas standardized sex differences were negligible for EDI-B and EDI-BD; sex-related differences were therefore not uniform across the three risk subscales. Exploratory interaction analyses did not provide multiplicity-adjusted evidence that the associations of age or body-fat percentage with BSQ or EDI-3 RF scores differed between boys and girls. None of the interaction terms reached nominal significance. Likewise, although several sex-stratified correlations reached nominal significance, none survived correction for multiple testing. These findings should therefore be considered exploratory signals requiring replication rather than confirmatory evidence of sex-specific associations.

The near-zero BSQ–EDI-BD association requires specific consideration because prior validation work has reported strong convergence between the BSQ and the Body Dissatisfaction scale of the Eating Disorder Inventory (r ≥ 0.72) [40], and a synthesis of 129 EDI-3 studies found generally robust, mostly large convergent correlations for the principal EDI-3 scales [41]. In the present study, participant identifiers, database linkage, item coding, missing-item rules, and reverse scoring were rechecked at the individual level, and no participant mismatches were identified. The observed coefficient therefore represents the analyzed sample, but it should not be interpreted as evidence that the instruments measure unrelated constructs.

Several sample and measurement features may have attenuated the association. Eligibility required at least 6 h of physical activity per week, which may have selected a relatively active and homogeneous school population; however, activity intensity and domain were not quantified, so this explanation cannot be tested directly. The BSQ also showed a marked concentration at the lower end: 105 of 723 participants (14.5%) had the minimum score of 34, and 237 (32.8%) scored below 40, with an observed range of 34–186. This floor concentration and the modest internal consistency of the BSQ (α = 0.71) and EDI-BD (α = 0.72) could have reduced covariance. These are plausible contributors rather than definitive explanations. The unexpected result remains sample-specific and should be replicated in studies designed to evaluate convergent validity across wider symptom ranges.

The between-sex BSQ difference was statistically significant and small in standardized magnitude (d = 0.21). Because no established minimal clinically important difference was applied, the clinical relevance of this difference cannot be determined from Cohen’s d alone. Peer comparison, sociocultural appearance norms, media exposure, pubertal timing, self-esteem, family context, and emotional regulation are plausible correlates described in the literature [41,42,43], but they were not measured in this study. They are therefore presented only as hypotheses for future research and not as explanations of the residual variance in the current models.

This study has several limitations. Its cross-sectional design precludes causal inference. Schools and participants formed a convenience sample from one Mediterranean city, limiting generalizability. The requirement of at least 6 h of physical activity per week may further limit applicability to less-active adolescent populations, and physical activity was not quantified beyond this eligibility criterion. Pubertal maturation was not assessed. BSQ and EDI-3 RF are self-report screening measures, not diagnostic instruments. Although participant matching, item coding, and questionnaire scoring were verified against the source data, the unexpected BSQ–EDI-BD correlation and inverse age coefficients may still reflect residual measurement error, range restriction, unmeasured developmental factors, or sample-specific characteristics and therefore require replication. Several body-composition variables were estimated from anthropometry; the Yuhasz equations were not developed specifically for a general adolescent population, and equation choice may influence the estimated values.

The current sample overlaps completely with two earlier reports from the same project, and the incremental contribution is confined to additional anthropometric/estimated body-composition variables and adjusted models. Strengths include the relatively large school-based sample, inclusion of both sexes, and simultaneous collection of anthropometric and questionnaire data.

## 5. Conclusions

In this convenience sample, girls had higher EDI-DT and BSQ scores, whereas EDI-B and EDI-BD showed negligible sex differences. The associations between EDI-3 RF scores and anthropometric or estimated body-composition indicators were small. Body-fat percentage was independently associated with greater EDI-DT scores after adjustment for age and sex, and with higher odds of an elevated BSQ score; its association with continuous BSQ scores was of borderline significance. Female sex was independently associated with higher Drive for Thinness scores, but not with BSQ scores after adjustment. Age was positively associated with BSQ but inversely associated with all three EDI-3 RF subscales. The near-zero BSQ–EDI-BD correlation cannot be interpreted as evidence of measurement non-equivalence and requires further evaluation. The findings are cross-sectional, sample-specific, and not causal.

## Figures and Tables

**Figure 1:**
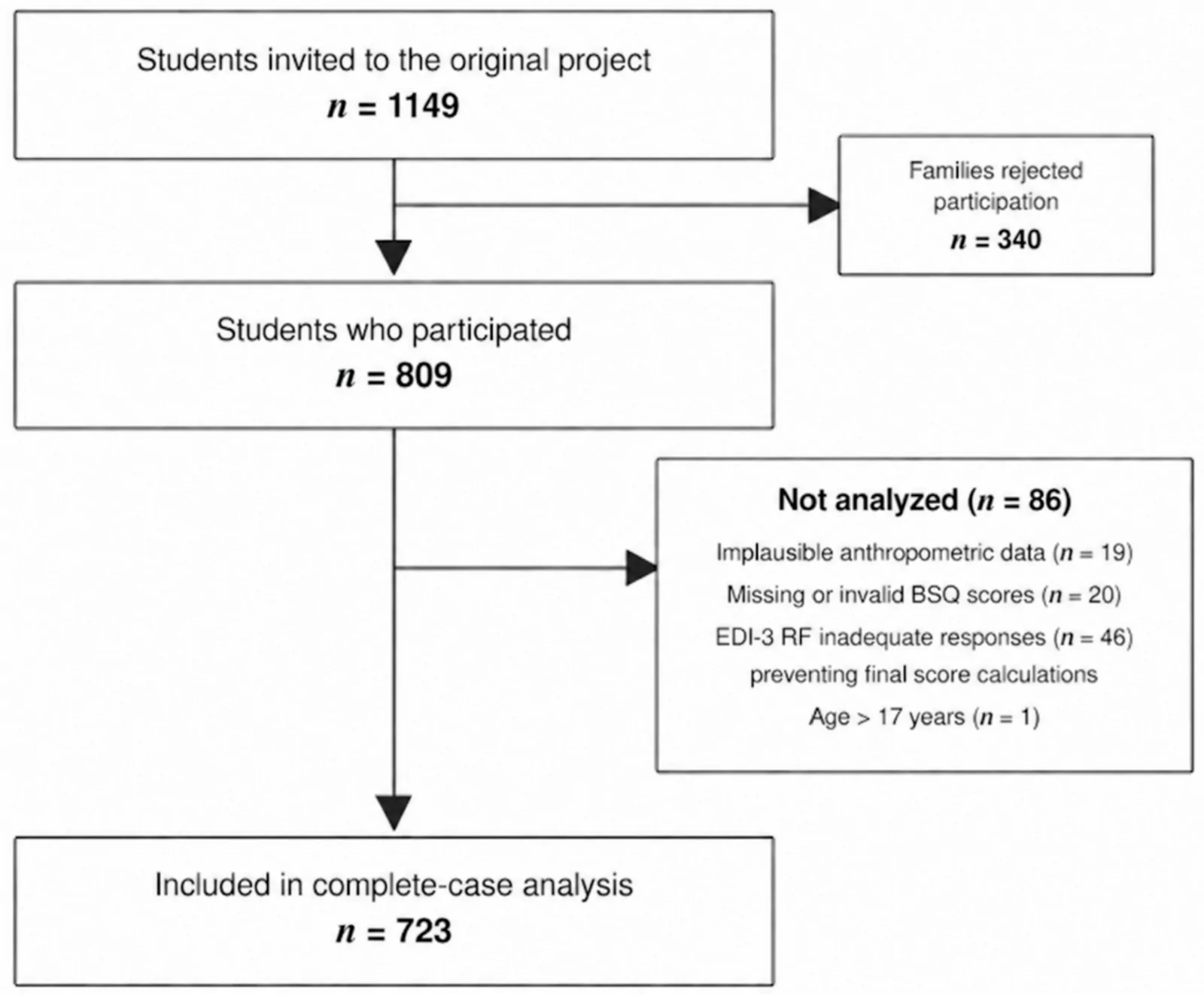
Participant flow diagram.

**Table 1:** Anthropometric characteristics and estimated body-composition indicators by sex.

Variable	Boys (*n* = 404)		Girls (*n* = 319)		Girls–Boys	*p*	Cohen’s d
	Mean (SD)	95% CI	Mean (SD)	95% CI	Mean Difference (95% CI)		
Age (years)	13.80 (2.05)	13.60 to 14.00	13.38 (1.91)	13.17 to 13.59	−0.42 (−0.71 to −0.13)	0.011	−0.21
Height (cm)	157.87 (10.94)	156.80 to 158.94	155.03 (8.54)	154.09 to 155.97	−2.84 (−4.27 to −1.42)	0.001	−0.29
Weight (kg)	50.98 (13.94)	49.62 to 52.34	47.32 (10.91)	46.12 to 48.53	−3.66 (−5.47 to −1.84)	<0.001	−0.29
BMI (kg/m^2^)	20.14 (3.47)	19.80 to 20.47	19.48 (3.08)	19.14 to 19.82	−0.66 (−1.13 to −0.18)	0.009	−0.20
Estimated body fat (%) ^a^	11.95 (4.75)	11.48 to 12.41	19.29 (6.24)	18.60 to 19.97	7.34 (6.51 to 8.17)	<0.001	1.34
Fat mass (kg) ^b^	6.45 (4.33)	6.02 to 6.87	9.61 (5.38)	9.02 to 10.20	3.16 (2.44 to 3.89)	<0.001	0.66
Lean mass (kg) ^b^	44.53 (10.70)	43.49 to 45.58	37.71 (6.72)	36.97 to 38.45	−6.82 (−8.10 to −5.54)	<0.001	−0.74
Muscle mass (kg) ^c^	24.82 (6.18)	24.21 to 25.42	21.24 (4.00)	20.80 to 21.68	−3.57 (−4.32 to −2.83)	<0.001	−0.67
Sum of six skinfolds (mm)	89.10 (45.24)	84.67 to 93.52	101.46 (40.30)	97.02 to 105.90	12.37 (6.11 to 18.62)	<0.001	0.29

Note: Values are mean (SD) and 95% confidence intervals (CIs) of the mean. Mean differences were calculated as girls minus boys; their 95% CIs were estimated using Welch’s method. *p*-values were obtained using two-sided Mann–Whitney U tests. Cohen’s d was calculated as girls minus boys using the pooled SD. BMI: body mass index. ^a^ Estimated with the sex-specific Yuhasz equations. ^b^ Fat mass = (body-fat percentage/100) × body weight; lean mass = body weight—fat mass. ^c^ Body weight minus fat, bone, and residual masses; *n* = 403 boys and *n* = 319 girls.

**Table 2:** BSQ and EDI-3 RF risk-subscale scores by sex.

Scale/Subscale	Boys (*n* = 404)		Girls (*n* = 319)		Girls–Boys	*p*	Cohen’s d
	Mean (SD)	95% CI	Mean (SD)	95% CI	Mean Difference (95% CI)		
BSQ	56.62 (27.19)	53.96 to 59.28	62.23 (26.71)	59.29 to 65.17	5.61 (1.65 to 9.57)	<0.001	0.21
EDI-3 RF—Drive for Thinness	8.36 (6.26)	7.75 to 8.97	11.43 (6.55)	10.71 to 12.15	3.07 (2.13 to 4.02)	<0.001	0.48
EDI-3 RF—Bulimia	5.29 (5.42)	4.76 to 5.82	5.61 (5.14)	5.04 to 6.18	0.32 (−0.45 to 1.10)	0.159	0.06
EDI-3 RF—Body Dissatisfaction	17.10 (8.55)	16.27 to 17.94	17.21 (6.68)	16.48 to 17.95	0.11 (−1.00 to 1.22)	0.572	0.01

Note: Values are mean (SD) and 95% confidence intervals (CIs) of the mean. Mean differences were calculated as girls minus boys; their 95% CIs were estimated using Welch’s method. *p*-values were obtained using two-sided Mann–Whitney U tests. Cohen’s d was calculated as girls minus boys using the pooled SD. BSQ: Body Shape Questionnaire; EDI-3 RF: Eating Disorder Inventory-3 Referral Form.

**Table 3:** Sex-stratified correlations of anthropometric indicators and BSQ scores with EDI-3 RF risk subscales.

Independent Variable	BSQ	EDI-3 RF—Drive for Thinness	EDI-3 RF—Bulimia	EDI-3 RF—Body Dissatisfaction
A. Boys (*n* = 404)				
BMI (kg/m^2^)	ρ = 0.133	ρ = 0.045	ρ = −0.096	ρ = −0.086
	95% CI 0.036 to 0.227	95% CI −0.052 to 0.142	95% CI −0.192 to 0.001	95% CI −0.182 to 0.012
	*p* = 0.008	*p* = 0.362	*p* = 0.054	*p* = 0.086
	q = 0.095	q = 0.723	q = 0.210	q = 0.297
Body fat (%)	ρ = 0.070	ρ = 0.137	ρ = −0.002	ρ = 0.059
	95% CI −0.028 to 0.166	95% CI 0.040 to 0.232	95% CI −0.100 to 0.095	95% CI −0.039 to 0.155
	*p* = 0.160	*p* = 0.006	*p* = 0.963	*p* = 0.239
	q = 0.468	q = 0.095	q = 0.963	q = 0.562
Muscle mass (%) ^a^	ρ = −0.065	ρ = −0.105	ρ = −0.019	ρ = −0.117
	95% CI −0.162 to 0.033	95% CI −0.201 to −0.007	95% CI −0.116 to 0.079	95% CI −0.212 to −0.019
	*p* = 0.194	*p* = 0.035	*p* = 0.704	*p* = 0.019
	q = 0.526	q = 0.210	q = 0.944	q = 0.181
Sum of six skinfolds (mm)	ρ = 0.070	ρ = 0.137	ρ = −0.002	ρ = 0.059
	95% CI −0.028 to 0.166	95% CI 0.040 to 0.232	95% CI −0.100 to 0.095	95% CI −0.039 to 0.155
	*p* = 0.160	*p* = 0.006	*p* = 0.963	*p* = 0.239
	q = 0.468	q = 0.095	q = 0.963	q = 0.562
BSQ	-	ρ = 0.057	ρ = 0.034	ρ = −0.013
		95% CI −0.041 to 0.154	95% CI −0.064 to 0.131	95% CI −0.110 to 0.085
		*p* = 0.252	*p* = 0.495	*p* = 0.799
		q = 0.562	q = 0.819	q = 0.949
B. Girls (*n* = 319)				
BMI (kg/m^2^)	ρ = 0.044	ρ = 0.112	ρ = −0.026	ρ = −0.011
	95% CI −0.066 to 0.153	95% CI 0.002 to 0.219	95% CI −0.135 to 0.084	95% CI −0.121 to 0.099
	*p* = 0.430	*p* = 0.046	*p* = 0.644	*p* = 0.843
	q = 0.777	q = 0.210	q = 0.944	q = 0.963
Body fat (%)	ρ = −0.006	ρ = 0.107	ρ = −0.020	ρ = 0.016
	95% CI −0.116 to 0.104	95% CI −0.002 to 0.215	95% CI −0.130 to 0.090	95% CI −0.094 to 0.126
	*p* = 0.916	*p* = 0.055	*p* = 0.720	*p* = 0.774
	q = 0.963	q = 0.210	q = 0.944	q = 0.948
Muscle mass (%) ^a^	ρ = 0.009	ρ = −0.126	ρ = −0.027	ρ = −0.042
	95% CI −0.101 to 0.118	95% CI −0.233 to −0.017	95% CI −0.137 to 0.083	95% CI −0.151 to 0.068
	*p* = 0.879	*p* = 0.024	*p* = 0.629	*p* = 0.454
	q = 0.963	q = 0.182	q = 0.944	q = 0.784
Sum of six skinfolds (mm)	ρ = −0.006	ρ = 0.107	ρ = −0.020	ρ = 0.016
	95% CI −0.116 to 0.104	95% CI −0.002 to 0.215	95% CI −0.130 to 0.090	95% CI −0.094 to 0.126
	*p* = 0.916	*p* = 0.055	*p* = 0.720	*p* = 0.774
	q = 0.963	q = 0.210	q = 0.944	q = 0.948
BSQ	-	ρ = −0.059	ρ = −0.045	ρ = 0.025
		95% CI −0.167 to 0.051	95% CI −0.154 to 0.065	95% CI −0.085 to 0.135
		*p* = 0.296	*p* = 0.426	*p* = 0.652
		q = 0.626	q = 0.777	q = 0.944

Note: Values are Spearman’s ρ with 95% confidence intervals (CIs) estimated using Fisher’s z transformation, followed by two-sided *p*-values and Benjamini–Hochberg-adjusted q-values across all 38 sex-stratified tests. Analyses included 404 boys and 319 girls, except those involving relative muscle mass, for which data were available for 403 boys and 319 girls. ^a^ Relative muscle mass was calculated as (muscle mass in kg/body weight in kg) × 100. Because the Yuhasz equations are linear functions of the sum of six skinfolds within each sex, the rank correlations for body-fat percentage and the sum of six skinfolds are identical within each sex.

**Table 4:** Sex × age and sex × body-fat percentage interaction terms added to the adjusted models (exploratory sensitivity analyses).

Outcome	Interaction Term	B	95% CI	*p*	q (BH)	ΔR^2^
BSQ	Sex × age	−1.334	−3.328 to 0.661	0.190	0.507	0.0082
BSQ	Sex × body fat (%)	−0.769	−1.699 to 0.161	0.105	0.421	
EDI-3 RF—Drive for Thinness	Sex × age	0.134	−0.365 to 0.634	0.598	0.683	0.0009
EDI-3 RF—Drive for Thinness	Sex × body fat (%)	−0.056	−0.237 to 0.125	0.545	0.683	
EDI-3 RF—Bulimia	Sex × age	0.159	−0.238 to 0.555	0.432	0.683	0.0009
EDI-3 RF—Bulimia	Sex × body fat (%)	0.018	−0.125 to 0.162	0.803	0.803	
EDI-3RF-Body Dissatisfaction	Sex × age	0.533	−0.063 to 1.128	0.080	0.421	0.0060
EDI-3 RF—Body Dissatisfaction	Sex × body fat (%)	−0.116	−0.328 to 0.095	0.281	0.561	

Note: *n* = 723. Age and body-fat percentage were mean-centered; boys were the reference category. Both interaction terms were added simultaneously to each model including sex, age and body-fat percentage. HC3-robust standard errors were used for the BSQ, EDI-DT and EDI-BD models. q-values: Benjamini–Hochberg adjustment across the eight interaction terms. ΔR^2^: increase in R^2^ after adding both interaction terms. Body-fat percentage was estimated with the sex-specific Yuhasz equations. BSQ: Body Shape Questionnaire; EDI-3 RF: Eating Disorder Inventory-3 Referral Form.

**Table 5:** Multivariable linear regression models for BSQ and EDI-3 RF risk-subscale scores.

Outcome	Predictor	B	95% CI	Standardized β	*p*
BSQ	Intercept	22.454	7.325 to 37.583	-	0.004
	Female sex (ref. boys)	3.373	−1.816 to 8.562	0.062	0.203
	Age (years)	2.107	1.121 to 3.092	0.155	<0.001
	Body fat (%)	0.426	−0.014 to 0.866	0.103	0.058
BSQ model	*n* = 723; R^2^ = 0.042; adjusted R^2^ = 0.038; F (3, 719) = 10.41 ^a^; *p* < 0.001				
EDI-3 RF—Drive for Thinness	Intercept	10.637	7.118 to 14.156	-	<0.001
	Female sex (ref. boys)	1.847	0.700 to 2.994	0.140	0.002
	Age (years)	−0.295	−0.534 to −0.055	−0.090	0.016
	Body fat (%)	0.150	0.061 to 0.239	0.150	<0.001
Drive for Thinness model	*n* = 723; R^2^ = 0.077; adjusted R^2^ = 0.073; F (3, 719) = 20.43 ^a^; *p* < 0.001				
EDI-3 RF—Bulimia	Intercept	9.300	6.455 to 12.146	-	<0.001
	Female sex (ref. boys)	0.327	−0.611 to 1.266	0.031	0.494
	Age (years)	−0.276	−0.470 to −0.082	−0.104	0.005
	Body fat (%)	−0.017	−0.087 to 0.054	−0.021	0.643
Bulimia model	*n* = 723; R^2^ = 0.012; adjusted R^2^ = 0.008; F (3, 719) = 2.90; *p* = 0.034				
EDI-3 RF—Body Dissatisfaction	Intercept	26.822	22.626 to 31.018	-	<0.001
	Female sex (ref. boys)	−0.580	−1.836 to 0.676	−0.037	0.366
	Age (years)	−0.748	−1.046 to −0.451	−0.192	<0.001
	Body fat (%)	0.051	−0.048 to 0.150	0.043	0.311
Body Dissatisfaction model	*n* = 723; R^2^ = 0.038; adjusted R^2^ = 0.034; F (3, 719) = 8.56 ^a^; *p* < 0.001				

Note: Predictors were entered simultaneously. Female sex was coded 1, with boys as the reference category. BSQ was analyzed as an outcome and was not included as a predictor. HC3-robust standard errors, confidence intervals, and *p*-values are reported for the BSQ, EDI-3 RF Drive for Thinness and EDI-3 RF Body Dissatisfaction models because heteroscedasticity was detected (Breusch–Pagan *p* ≤ 0.005); conventional ordinary least-squares estimates are reported for the Bulimia model (Breusch–Pagan *p* = 0.155). ^a^ Robust (HC3) Wald F-test. VIF ranged from 1.01 to 1.46 and tolerance from 0.68 to 0.99. BSQ: Body Shape Questionnaire; CI: confidence interval; EDI-3 RF: Eating Disorder Inventory-3 Referral Form; VIF: variance inflation factor.

**Table 6:** Multivariable logistic regression for an elevated BSQ score (≥105 points).

Predictor	Adjusted OR	95% CI	*p*
Body fat (per 1% increase)	1.053	1.007 to 1.100	0.023
Age (years)	1.221	1.062 to 1.404	0.005
Female sex (ref. boys)	1.073	0.552 to 2.085	0.836

Note: The binary outcome was coded 0 for BSQ <105 and 1 for BSQ ≥105 [26]. The model included 723 adolescents, of whom 55 (7.6%) had an elevated BSQ score. Predictors were entered simultaneously. Female sex was coded 1, with boys as the reference category. Body-fat percentage was estimated with the sex-specific Yuhasz equations. BSQ: Body Shape Questionnaire; CI: confidence interval; OR: odds ratio.

## Data Availability

Raw data are available upon request to corresponding author.

## Abbreviations

The following abbreviations are used in this manuscript:

BMI	Body mass index
ED	Eating disorder
EDI-3	Eating Disorder Inventory-3
EDI-DT	Eating Disorder Inventory-3 Drive for Thinness
EDI-B	Eating Disorder Inventory-3 Bulimia
EDI-BD	Eating Disorder Inventory-3 Body Dissatisfaction
BSQ	Body Shape Questionnaire
